# Buffalo Academy of Medicine

**Published:** 1895-01

**Authors:** E. J. Gilray

**Affiliations:** Secretary


### BUFFALO ACADEMY OF MEDICINE—SURGICAL SECTION.

Reported by E. J. GILRAY, M. D., Secretary.

The regular monthly meeting of the Surgical Section of the Academy of Medicine was held at the Academy parlors, November 6, 1894, Dr. Hartwig presiding. Present—Drs. Grover Wende, Van Peyma, Rochester, Mynter, Shroetter, Horace Clark, Cott, Grosvenor, Hinkel, Rogers, Hubbell, Krauss, Bennett.

Dr. Gilray exhibited a case of syphilis of the throat.

Dr. Cott read a paper on Surgery of the Accessory Sinuses (page 321 of the Journal).

Dr. Horace Clark, in opening the discussion, thought that surgery of the accessory sinuses was the most difficult in the field of nasal surgery. The surgical treatment of diseases of the ethmoidal and sphenoidal sinuses was almost impossible, being both difficult and dangerous. He thought that differential diagnosis between ethmoidal and sphenoidal disease could be made more easily from symptoms than from location of pus. The removal of hypertrophic tissue and polypi is not necessary in treatment. He thought the introduction of a tube through the tooth cavity was unnecessary when an opening could be made through the nasal wall.

Dr. Mynter exhibited some new drills for opening into the antrum of Highmore through the tooth cavity.

Dr. Hinkel: The treatment of the accessory sinuses may be divided into the treatment of the antrum and the other three sinuses. He could not agree with Dr. Clark's statement that ethmoidal disease was rare. From private statistics, since 1889, he has had thirty cases of disease of the antrum. Of these, twenty-one were chronic and nine were acute. In the twenty-one chronic there were polypi in ten cases; in the remaining eleven there were carious teeth in seven. These thirty cases comprise about two per cent. of all his cases. Of ethmoidal disease, during the same length of time, he had seen nineteen cases, of which fifteen were chronic and four acute. Of the fifteen chronic cases there were polypi in eight. The percentage of ethmoidal disease, therefore, in his practice was about 1.2 per cent. Of sphenoidal disease he had suspected three cases. Of frontal abscess he had seen two cases. He did not think it so difficult to open into the sphenoidal and ethmoidal cells. In ethmoidal disease he removes the anterior end of the middle turbinated body; then, by means of a curette and drill, breaks up the cells and gives fairly good drainage. The results are not always successful. He never had to perform an operation in frontal abscess.

Secure a large opening in antral disease for drainage. Recent cases of antral abscess are very amenable to treatment. If a tooth is at all sensitive, he believes in sacrificing the tooth, then drilling and draining through the tooth cavity. It is true the treatment of the accessory sinuses is not yet satisfactory. The tendency should be for larger openings and bolder surgery.

Dr. Cott, in reply to Dr. Clark about location of pus, had spoken about it after mentioning other symptoms. He is positive that many cases of sphenoidal and ethmoidal disease do not get well.

Dr. Grover Wende presented a short paper on An Unusual Case of Herpes.

Dr. Clark reported a case of primary tuberculosis of the fauces.

The meeting adjourned at 10.30 p. m.

